# Predictors of default from follow-up care in a cervical cancer screening program using direct visual inspection in south-western Nigeria

**DOI:** 10.1186/1472-6963-14-143

**Published:** 2014-03-31

**Authors:** Oliver Chukwujekwu Ezechi, Karen Odberg Petterson, Titilola A Gabajabiamila, Ifeoma Eugenia Idigbe, Olutunmike Kuyoro, Innocent Achaya Otobo Ujah, Per Olof Ostergren

**Affiliations:** 1 Clinical Sciences Division, Nigerian Institute of Medical Research, Lagos, Nigeria; 2 Social Medicine and Global Health, Faculty of Medicine, Lund University, Lund, Sweden

**Keywords:** Cervical cancer, Direct visual inspection, VIA, VILI, Default

## Abstract

**Background:**

Increasingly evidence is emerging from south East Asia, southern and east Africa on the burden of default to follow up care after a positive cervical cancer screening/diagnosis, which impacts negatively on cervical cancer prevention and control. Unfortunately little or no information exists on the subject in the West Africa sub region. This study was designed to determine the proportion of and predictors and reasons for default from follow up care after positive cervical cancer screen.

**Method:**

Women who screen positive at community cervical cancer screening using direct visual inspection were followed up to determine the proportion of default and associated factors. Multivariate logistic regression was used to determine independent predictors of default.

**Results:**

One hundred and eight (16.1%) women who screened positive to direct visual inspection out of 673 were enrolled into the study. Fifty one (47.2%) out of the 108 women that screened positive defaulted from follow-up appointment. Women who were poorly educated (OR: 3.1, CI: 2.0 – 5.2), or lived more than 10 km from the clinic (OR: 2.0, CI: 1.0 – 4.1), or never screened for cervical cancer before (OR: 3.5, CI:3:1–8.4) were more likely to default from follow-up after screening positive for precancerous lesion of cervix . The main reasons for default were cost of transportation (48.6%) and time constraints (25.7%).

**Conclusion:**

The rate of default was high (47.2%) as a result of unaffordable transportation cost and limited time to keep the scheduled appointment. A change from the present strategy that involves multiple visits to a “see and treat” strategy in which both testing and treatment are performed at a single visit is recommended.

## Background

Every year half a million women are diagnosed with cervical cancer and another 250,000 reported dead from the disease, which makes cancer of the cervix the second most common female cancer globally [1-3]. It is thus a major public health challenge especially in resource-poor settings of sub Saharan Africa and south east Asia where it accounts for most of the morbidity and mortality attributable to cancer in women [4,5]. In these settings, over 80% of cases of cervical cancer presents in the late stage when very little can be done [4,5]. Late-stage disease is associated with low survival rates even after the use of surgery and/or radiotherapy. In addition, these treatment modalities may be lacking altogether, or be too expensive and inaccessible for many women in low-resource settings.

Cervical cancer is potentially preventable, and effective screening programmes can lead to a significant reduction in the morbidity and mortality it causes [3,6,7]. In sub-Saharan Africa, there are few organized efforts to ensure that women over the age of 30 years are screened [6,8]. Consequently women with cervical cancer are not identified until they are at an advanced stage of disease, resulting in high morbidity and mortality [9].

The introduction of cytology based screening programs to women in all populations in high income countries has been shown to reduce cancer of the cervix rates by 60–90% within three years of implementation [10,11]. While the high-income countries have successfully used this method to reduce the burden and deaths associated with cervical cancer, most low income countries have not succeeded in a similar manner due to inadequate human and infrastructural resources [3,4]. This challenge prompted the introduction and adoption of the low cost technology screening programme based on direct visual inspection (DVI) of the cervix in sub-Saharan Africa and south east Asia [10,12-14]. While countries such as India, Thailand and Zambia [15-17] have implemented this strategy with a positive impact both on the burden and the mortality, several nations including Nigeria are yet to implement the strategy successfully [13,18-21].

In Nigeria, cervical cancer screening services using visual inspection is not yet institutionalised despite its adoption by the Nigerian government as the prevention and control strategy of choice [12,22,23]. The cervical cancer screening is largely opportunistic during gynaecological consultations and uncoordinated outreach programmes often performed by nurses and medical officers [12,20]. Moreover women found positive at the outreach programmes are referred for confirmation at a tertiary facility several kilometres away [20,24]. There is consequently a risk of drop-out in such a system, as the women have to make multiple visits for confirmation of screening result and treatment [23-27].

In low-income countries, returning for follow-up care after screening positive can be a challenge for women due to sociocultural, financial, practical, and logistical barriers. Others may not return for follow-up visits because they do not properly understand the importance of further evaluation or are afraid of receiving bad news about their condition. Others may not return due to embarrassment or a fear of diagnosis or treatment as reported by researchers in Nigeria, England and Uganda [22,25-29]. Increasingly evidence is emerging from south east Asia and sub-Saharan Africa on the burden of non-adherence to follow-up care after a positive cervical cancer screening/diagnosis and its impact on cervical cancer control in low and medium income countries [25-28]. Default rates in programs designed to identify cervical cancer at precancerous stages are usually very high, ranging from 5-20% in high income countries and 20-41% in low income countries [24,29-32].

Although there are studies from our sub-region reporting on default in pap smear based screening programmes [18,20-22], detailed literature search showed few studies reporting the level of default and the reasons for it after screening positive to DVI during community outreach cervical cancer screening [24].

In June 2011, the Nigerian Institute of Medical Research (NIMR) in Lagos, the largest city in Nigeria, initiated a community based outreach cervical cancer screening programme as a corporate social responsibility after successfully integrating cervical cancer screening into its HIV care services [23]. However, few months into the outreach services, a substantial default was noted from the follow-up appointment for women that had tested positive. The need to confirm this preliminary finding prompted this study, which is a part of a larger study on the interaction between HIV, HPV and cervical premalignant lesions. Information obtained may assist to improve the quality of the programme and other similar programmes [10,12,18].

The overall aim of this study was to investigate the magnitude of default and factors associated with default from follow-up care after screening positive to cervical precancerous lesion using direct visual inspection.

## Methods

### Study setting

Four urban and 6 rural communities in the south western Nigerian states of Lagos and Ogun that participated in community outreach cervical cancer screening programme conducted by NIMR between October 2011 and December 2012 served as the setting. While three urban (Surulere, Ikeja and Gbagada) and 4 rural (Mushin, Iju, Ikorodu and Egbeda) communities were in Lagos State, the remaining urban (Sagamu) and 2 rural (Ifo and Ibafo) communities were in Ogun state. The average distance of the communities to the NIMR ranged from 2 to 70 kilometres. While the study participants from the rural communities were largely poor, poorly educated and of low socioeconomic status, those from the urban communities were comparatively of higher educational and socioeconomic status. However in the urban communities, pockets of urban slums exist side by side with areas of affluence. This is often the pattern in most cities in Nigeria and other west African countries. It is also important to note that though the communities are in two states, there is no clear demarcation between the states as rapid urbanisation had led to the merger of border communities in the two states.

The Nigerian Institute of Medical Research in Lagos is the apex medical research Institution in Nigeria and an agency of the Federal Ministry of Health charged with the responsibility to conduct research on diseases of public health importance in the country.

### Study design

#### Prospective study design

### Study population

Adult women of known HIV status aged 18 years and above who screened positive or inconclusive to direct visual inspection of cervix during community outreach cervical cancer screening programme and consented to be part of the study were enrolled into the study. Enrolment for this specific study commenced on the 2nd October 2011 and follow up completed on the 17th December 2011.

Before the screening proper the women were educated as a group on cervical cancer screening and the importance of keeping follow-up appointment if found positive using study informed consent document approved by the ethics committee. It addressed the following issues;

● What is cervical cancer and its premalignant phase.

● Various strategies for the prevention of cervical cancer.

● What is cervical screening and why they need to do the screening?

● Who should have the test?

● The Nigerian cervical cancer screening Programme and the step in arriving at a definitive diagnosis?

● How reliable is cervical screening?

● Who will carry out the test and how it will be conducted?

● Follow up schedules after screening.

The main benefits and difficulties of cervical screening are explained.

### Clinical procedures

After signing the Informed Consent Form, information on sociodemographic characteristics, sexual and reproductive history were collected using study case record form. All participants were subjected to a thorough pelvic examination, in this sequence comprising collection of the Pap smear, collection of sample for microbiological examination (when indicated) and Direct Visual Inspection (DVI) using either Acetic Acid (VIA) or Lugol’s Iodine (VILI). The examinations were performed by physicians and midwives received training before performing the study procedures.

The women were placed in modified lithotomy position and the cervix is exposed with the help of a Cusco’s bivalve speculum and examined.

Cervical scraping is obtained by placing the Ayres spatula at the cervical os and rotated gently by 360° twice. A Smear was prepared by spreading the specimen uniformly across a pre-labeled glass slide, which was immediately fixed using a commercial fixator containing 95% ethyl alcohol.

After collecting the cervical smear, the same examiner performed VIA or VILI depending on a predetermined group allocation.

### VIA procedure and interpretation

After collection of the samples for the Pap test and microbiological test, acetic acid was applied to the cervix through embedded cotton at the edge of a sponge holding forceps. After 1 min, the cervix was illuminated with a bright lamp and visually examined (‘naked eye’ examination). Examiners have been trained on how to classify their visual impression. For statistical purposes, these impressions were grouped as negative or positive. The findings of VIA were recorded using the following criteria:

#### VIA negative

When any of the following findings were observed: no acetowhite lesions; faint, ill-defined, bluish white, shiny, translucent or doubtful acetowhite lesions; dot-like or streak-like acetowhitening in the columnar epithelium; white-line like prominent squamocolumnar junction; acetowhitening on endocervical polyps or on nabothian cysts; or acetowhite lesions distant from the squamocolumnar junction.

#### VIA positive

When any of the following findings were observed: well-defined, demarcated, opaque, acetowhite lesions with or without raised margins, touching or close to the squamocolumnar junction well-defined, dense circumorificial acetowhite lesion involving all the four quadrants of the cervix; condylomata and leukoplakia occurring close to the squamocolumnar junction turning intensely white after the application of acetic acid; or dense, opaque, acetowhite lesions on clinically visible ulceroproliferative growth of the cervix.

### VILI procedure and interpretation

After collection of the samples for the Pap and microbiological test, the cervix was stained with Lugol’s iodine. The cervix was illuminated with a bright lamp and visually examined. Examiners have been trained on how to classify their visual impression. For statistical purposes, these impressions were grouped as negative or positive. The findings of VILI were recorded using the following criteria.

#### VILI negative

Homogeneous staining of the cervix was obtained after application of Lugol’s iodine.

#### VILI positive

A well-delimited non-staining area was present.

### Follow up

After the screening the women were appointed to visit cervical cancer screening clinic at NIMR for detailed counselling on the outcome of their test and further management either in the same or the following week. Women who did not honour the invitation after two weeks were contacted by phone or visited at home to ascertain the reason(s) for not keeping their appointment. The women we were able to reach were given another suitable date for the follow-up visit. The ones who failed to keep the second appointment or were not reached were considered defaulters.

All the women who screened positive to DVI during the community outreach services constitute the study population.

### Sample size determination

The sample size for this study was based on a reference VIA positive rate obtained in Sagamu Nigeria during the WHO demonstration project in six African countries (WHO) of 5.7% [33]. The study sample size was calculated according to the following formula: N = Zα2P (1-P)/d2, where Zα is the Z statistic for a 95% confidence level, N is the sample size, p is the VIA positive rate, and d is the precision [34]. Based on this calculation, following up 72 DVI positive women aged 18 years and above was considered sufficient to identify defaulters. The sample size was however increased by 25% in anticipation of non-acceptance to be followed up after testing positive to DVI. A final minimum sample size of 90 was obtained.

### Data collection

Participants who did not keep their appointments were contacted on the phone or visited at home and rescheduled for another appointment at a mutually acceptable convenient time. Participants who missed the rescheduled appointment were interviewed to obtain quantitative data on reason(s) for default using study case record forms.

### Definition of variables

● Default from follow-up: Women who screened positive but failed to keep the rescheduled second appointment or could not be reached. Women who screened positive and presented at another health facility for confirmation and or treatment are not included.

● Age: Age at the last birthday.

● Parity: Number of previous births after 28 weeks of gestation.

● Educational Status: The educational level completed by the women.

● Occupation: The main income generating activity/work engaged in by the women.

● Type of community: The communities were classified into urban or rural depending on government classification of the community.

● Marital status: The current marital status of the women (married, divorced, single or widowed)

● Distance of community to clinic: The approximate distance in kilometres from the women’s community to NIMR clinic.

● Current use of contraceptive: women who were using a modern method of contraceptive at the time of study.

● Previous cervical cancer test: Women who had previously tested for cervical cancer irrespective of method.

### Data analysis

Information on the case record forms were coded and analyzed using the SPSS version 19.0 (SPSS Inc. Chicago, IL) statistical packages. The main outcome variable was default from follow-up care after a positive screen to DVI. The outcome variable was examined against a set of independent variables (Sociodemographic, reproductive and residency characteristics) in order to determine factors that were associated with default to follow-up care. Bivariate analysis involved the use of Chi-squared tests to assess the significance of associations. Multivariate logistic-regression models were used to identify predictors of default from follow-up care. Because the number of events was small for prediction, we sought to minimize over fitting of the models by restricting the selection of variables to those that had previously been identified as relevant based on clinical experience and from literature. Candidate variables for the model included age, educational status, previous cervical cancer screening, distance from residence to health facility and type of residence. The odds ratios with 95% confidence intervals were calculated for each variable.

### Ethical considerations

Approval for the study was obtained from the Institutional Review Board, Nigerian Institute of Medical Research, Lagos Nigeria. A written informed consent was obtained from the women invited to be part of the study after detailed information about the study. Impartial witnesses who were not members of the team assisted the consenting process for the low literates.

## Results

**Figure 1:**
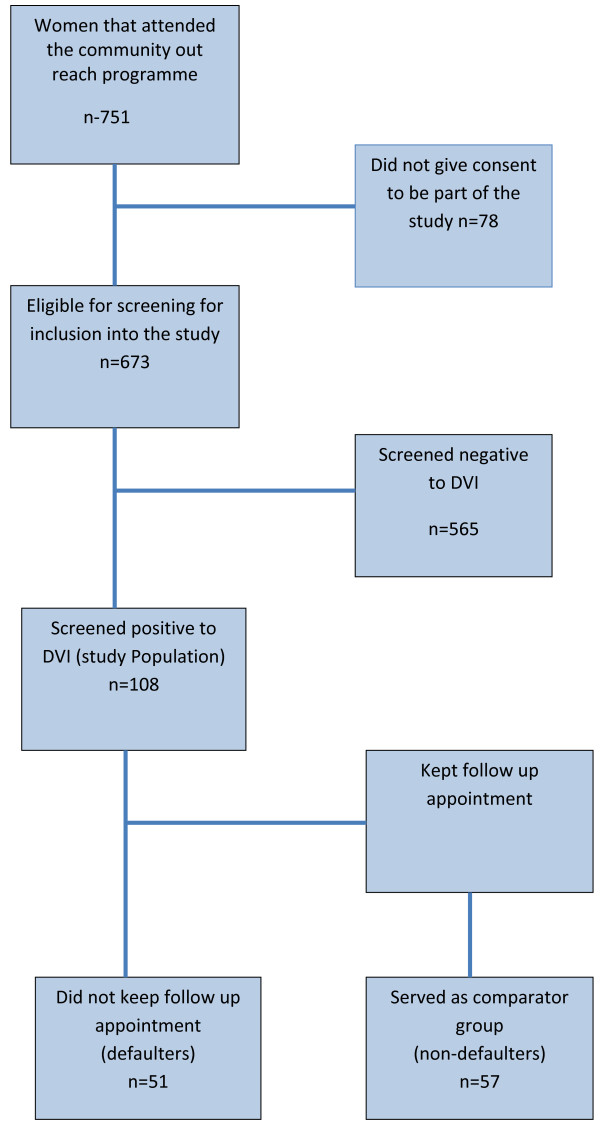
Study participation and follow up status.

A total of 751 women attended the outreach programme during the study period; of which 673(89.6%) consented to be part of the study. A total of 108 (16.1%) were found positive to direct visual inspection and constituted the study population (Figure 1). Forty (6.4%) of the 629 women that accepted to be screened for HIV tested positive. Of the forty, only seven screened positive to DVI.

### Sociodemographic and reproductive characteristics

The socio-demographic and reproductive characteristics of the 108 women who screened positive to DVI are presented in Table 1. Seventy four (68.5%) participants resided in a rural community compared to 34 (31.5%) that lived in an urban community. The approximate distance of the communities to the clinic ranged from 2 km to around 70 km, with the majority residing within 20 km of the clinic (63.9%). Only 12.0% of the women resided more than 50 km from the clinic. The mean age of the women was 39 ± 7 years (range 18–76). The majority of the participants were between 30 to 49 (65.7%) years of age. Married women (72.6%) and those of parity 1–4 (64.3%) were in the majority. Seventy eight (72.2%) women had completed at least secondary level education, 19 (17.6%) had completed primary education and 10 (9.3%) women had no formal education. The majority of the 108 women engaged in income generating activities (98; 90.7%), did not use a modern contraceptive method (76; 70.4%) and had not been subjected to cervical cancer screening prior to present screening (86; 79.6%).

**Table 1:** Socio-demographic and reproductive characteristics of the 108 women who screened positive to direct visual inspection

**Characteristics (valid data)**	**Number of participants (%)**
**Age (107)**	
18 -20	1(0.9)
20-29	16(15.0)
30-39	28(26.2)
40-49	43(40.2)
≥ 50	19(17.8)
**Parity (101)**	
0	9(8.9)
1-4	79(78.2)
≥ 5	13(12.9)
**Educational status (107)**	
None	10(9.3)
Primary	19(17.8)
Secondary	38(35.5)
Tertiary	40(37.4)
**Occupation (106)**	
Unemployed	29(27.4)
Unskilled	32(30.2)
Skilled	35(33.0)
Business executive/Professional	11(10.4)
**Marital status (107)**	
Single	14(13.1)
Married	77(72.0)
Divorced/Separated	4(3.7)
Widowed	9(8.4)
**Type of community (108)**	
Urban	34(31.5)
Rural	74(68.5)
**Distance of the communities to clinic (108)**	
<5	14(13.0)
5-10	22(20.4)
11-20	31(28.7)
21-50	28(25.9)
≥50	13(12.0)
**Current use of contraceptive (108)**	
Using	32(29.6)
Not using	76(70.4)
**Previous cervical cancer test (108)**	
Screened	22(20.4)
Not screened	86(79.6)
**HIV status**	
Positive	7(6.5)
Negative	101(93.5)

### Pattern of and reasons for default

Fifty-one (47.2%) women out of the 108 that tested positive to DVI defaulted from the scheduled follow-up care appointment. Of the 51 defaulters, fifteen (29.4%) were completely lost as both their phone number and home addresses were incorrect and therefore could not be located. The reasons for default among the 36 women that were reached were unaffordability of transportation and treatment cost (48.6%), time constraint (25.7%), ‘I believe nothing is wrong with me’ (11.4%), ‘the clinic is too far from my house’ (8.6%) and ‘I am sick’ (5.7%).

### Predictors of default from follow-up care

The results of the association between the outcome variable “default from follow-up care” and the socio-demographic and reproductive characteristics are shown in Table 2. A greater proportion of women who defaulted from follow-up care after screening positive to DVI had less than secondary education (OR: 2.9,95% CI: 1.1 –7.7) and lived in a rural community (OR: 2.78, CI:1.1 – 7.3) Women who resides more than 10 km from the clinic (OR: 3.6, CI:1.5 – 9.1), and had never screened for cervical cancer (OR: 3.9, CI:1.2–13.4) were in the majority compared to their counterparts who reside less than 10 km from the clinic and had screened for cervical cancer previously. No significant association was found between age, parity, marital status, work status, current contraceptive use and default from follow-up care. After controlling for confounders of educational status, work status, type of community and current contraceptive use in a multivariate logistic regression model, only 3 variables retained their independent significant association with default from follow up care appointment. These variables were having less than secondary education (OR = 3.1, CI: 2.0-5.2), residing more than 10 km from the clinic (OR: 2.0,95% CI: 1.0-4.1) and no previous cervical cancer screening experience (OR: 3.5, 95% CI: 3.1-8.4).

**Table 2:** Association between default from follow-up care after screening positive to precancerous lesion of the cervix and sociodemographic and reproductive characteristics of the participants (n = 108)

**Characteristics**	**Defaulter (%) n = 51**	**Non defaulter (%) n = 57**	**Crude result**		**Adjusted result**	
			**Crude OR (95% CI)**	**P value**	**Adjusted OR (95% CI)**	**P value**
**Age (years**)						
<40	25(49.0)	20(35.1)	1.8(0.8 – 4.2)	0.20	1.2(0.8 – 6.9)	0.23
≥40	26(51.0)	37(64.9)	1(ref)		1(ref)	
**Parity**						
≤2	15(29.4)	18(31.6)	2.0(0.8 – 5.6)	0.88	1.1(0.5 – 8.1)	0.95
>2	3(70.6)	35(68.4)	1(ref)		1(ref)	
**Education status**						
< Secondary	19(37.5)	10(17.5)	2.9(1.1 – 7.7)	0.03	3.1(2.0 – 5.2)^a^	0.02
≥ Secondary	31(62.7)	47(82.5)	1(ref)		1(ref)	
**Marital Status**						
Not married	14(27.5)	9(15.8)	2.0(0.7 – 5.6)	0.23	1.3(0.8 – 6.9)	0.26
Married	37(72.5)	47(84.2)	1(ref)		1(ref)	
**Work status**						
Not working	27(52.9)	19(33.3)	2.3(1.0 – 5.4)	0.06	1.2(0.8 – 8.1)	0.11
Working	23(47.1)	37(66.7)	1(ref)		1(ref)	
**Type of Community**						
Urban	10(19.6)	23(40.4)	1(ref)	0.03	1(ref)	0.07
Rural	41(80.4)	34(59.6)	2.8(1.1 – 7.3)		1.6(0.9 –9.1)	
**Distance of residence to clinic**						
< 10 Km	11(21.6)	25(43.9)	1(ref)	0.02	1(ref)	0.03
≥ 10 Km	40(78.4)	32(56.1)	2.8(1.1 – 7.3)		2.0(1.0 –4.1)^b^	
**Current contraceptive use**						
Using	10(19.6)	22(38.6)	1(ref)	0.05	1(ref)	0.08
Not using	41(80.4)	35(61.4)	2.6(1.0 -6.8)		1.4(0.3 – 9.7)	
**Previous cervical cancer screen**						
Screened	5(9.8)	17(29.8)	1(ref)	0.02	1(ref)	
Never Screened	46(90.2)	40(70.2)	3.9(1.2 –13.4)		3.5(3.1 – 8.4)^c^	0.006
**HIV status**						
Positive	3(5.9)	4(7.0)	0.8(0.1-4.7)	0.66	0.8(0.2-5.1)	0.81
Negative	48(94.1)	53(93.0)	1(ref)		1(ref)	

Note: Potential confounders adjusted at multivariate logistic regression analysis. ^a^Adjusted for work status, ^b^Adjusted for type of community and work status, ^c^Adjusted for educational status and current contraceptive use.

## Discussion

While some studies have evaluated the DVI test outcome, there is limited knowledge regarding default of follow-up care and associated factors after screening positive to cervical cancer [25-28]. This study was designed to fill this knowledge gap and to use the information obtained as advocacy tool for the improvement of the current opportunistic and outreach based cervical cancer screening in our sub-region. Overall 108 (16.0%) of 673 women tested positive to DVI; of which 47.2% defaulted from follow-up care appointment. The default rate in this study is much higher than rates reported from other low income countries and may be due to differing sociocultural, environmental and health system issues [5,18,23]. In Nigerian women are social-culturally expected to be subservient to their spouse and thus needs their permission to honour invitations including hospital visits [23,35]. In addition, a large percentage of women especially in rural areas are not gainfully employed depending on their spouse for their daily sustenance. Even transportation fees are provided by their spouse and thus need his concurrence to attend clinic for follow up. Poor public transportation and health systems are other challenges confronting the women and making it difficult to honour such invitation [18,23]. A large percentage of Nigerian women prefer to present in private health facilities because of the long waiting time and poor quality of services in public facilities [36,37]. Cost of transportation is also out of reach for poor peasants in the rural communities [36]. In the final analysis the choice the women make will depend on having to choose between keeping an appointment for a “disease they are not aware off” to “disobeying” their spouses with its possible consequences including violence [32,38]. The women are also mindful of the fact that in a country with poor health system and its attendant long waiting time, keeping appointment do not equate to finding solution to their “health challenge”. Furthermore the encroachment of the churches and mosques in health care without providing health infrastructure like the traditional mission hospitals could be a contributor [35]. In our earlier study on the willingness to screen for cervical cancer, the second most common reason for refusal of the test was related to client’s religious belief and teaching [23]. Some clients were of the opinion that they cannot have cervical cancer because of their religious belief. It may therefore be correct to assume that some participants in this study with similar belief may have defaulted.

Cervical cancer prevention programme that adopts a “see and treat” strategy could best suit the women in the setting described above. Several studies in sub Saharan African that evaluated the “see and treat” strategy reported very good outcome with significant reduction in default rate [26,39-42] The reduction in default rate was attributed to the reduction in number of visits, reduced service and transportation costs as well reduction in man hours of work [39-41].

Poorly educated women, living more than 10 km from the clinic and had never screened for cervical cancer in this study were more likely to default from follow-up care appointment than their more educated counterparts, residing less than 10 km from the clinic and had previously screened for cervical cancer. The main reasons for default were investigated among the defaulting women that could be reached (about half) and the main reasons given were unaffordability of transportation and treatment cost (48.6%), time constraints (25.7%) and ‘I believe nothing is wrong with me”.

The DVI positivity rate among the women in the study is 16.1% and thus within the range of 2% to 16% reported by studies conducted in Tanzania, Mali and Botswana [39,43,44]. It is also lower than 32.6% and 43.7% reported from South Africa and Zambia, respectively [45,46]. The lower rate in our study may be due to the lower HIV prevalence in the study communities compared to South Africa and Zambia [29]. A number of studies in sub Saharan Africa have reported a significant association between HIV infection and invasive cervical cancer [18,23,24,27,39,44,47]. In the presence of HIV infection, there is the persistence of cervical cancer-causing Human Papilloma Virus in the uterine cervix. The persistence of high-risk HPV types in the transformation zone of the cervix ultimately leads to malignant changes in the cervix [1,9,18].

The proportion of women who tested positive to DVI and did not attend the follow-up care appointment in this study (47.2%) is higher compared with findings from Zambia (40.8%) [29], and from Cote d’Ivoire (36.5%) [24]. The high default rate across the three studies most likely underscores the general lack of awareness and poor knowledge of cervical cancer and the importance of early diagnosis and treatment of cervical cancer among women in sub Saharan Africa [18,20,23]. Efforts are required to sensitize women to the serious consequences of untreated cervical cancer and the advantages of an early identification of premalignant lesions of the cervix. In addition, interventions should, besides screening, also include follow-up care through the process until treated or discharged from the clinics. Several countries in sub Saharan African and Southeast Asia have adopted the “see and treat strategy” in order to ensure prompt treatment and consequently prevention of default, a strategy found to be effective also in the hands of nurses and clinical officers [10,25,28]. Besides reducing cost and saving lives it will also address the reasons for default from follow-up care provided by the participants namely transportation cost and time constraints. This will most likely encourage more women to take the test, as it will eliminate repeat visit and long waiting time in public institutions.

The study was also able to establish the predictors for follow-up care default among women that screened positive to DVI. Those with poor educational status, residing more than 10 km from the clinic and who had not screened previously were more likely to default from follow-up care visit. This finding is in keeping with several studies in Nigeria and elsewhere, which show that poorly educated women and those who have never accessed reproductive health services are very likely to default from accessing other reproductive health services [18,20,23,24,27]. Poorly educated women may not be aware of the importance of positive cervical cancer screening and the follow-up visits. In addition, they are likely not to be gainfully employed, thus depending on spouses to finance all their needs including reproductive health services. The fact that apart from screening, all other services relating to confirmation and treatment of cervical cancer are not free may have demotivated the women from keeping the appointment. After all it may appear pointless spending limited resources to keep the appointment to women who may not afford the definitive treatment if confirmed positive.

The distance from the community to the clinic and the time spent travelling and waiting for services is another barrier found to keep women from follow-up appointment [18,23,24,28]. This is similarly confirmed by this study which showed that women who resided more than 10 km from the clinic were twice likely to default than those who reside within 10 km of the clinic.

Findings from this study confirmed our suspicion that significant numbers of women do not keep their follow-up appointment after screening positive to precancerous lesion. The reasons of cost of transportation and lack of time to keep the appointment are in keeping with findings in previous studies conducted in Ivory coast, Thailand, Mexico and Zambia for non-uptake of reproductive health services [4,5,15,16,24]. The findings confirm the long known fact that service provision alone is not sufficient for services uptake [5]. Rather issues of geographic access, low status of women, inadequate or lack of education and poverty are the major determinants of service uptake [6,14,15]. The factors found to be associated with default and reasons for defaults in this study confirm this. Female education and empowerment, community involvement and provision of cervical cancer services in the community are therefore the likely keys to the reduction of default form cervical cancer services [20,23,44].

### Limitations of the study

The number of events in this study was small and there was the likelihood of over fitting the model during analysis. We minimized this by restricting the selection of variables to only those that had been identified as relevant based on clinical experience and previous literature.

## Conclusion

The findings from this study contributed to the growing body of evidence indicating that the current strategy of opportunistic testing and outreach cervical cancer screening programme is associated with high rates of default. The multiple visits associated with this strategy and cost implication makes it burdensome and unattractive to poorly educated women residing several kilometres from the few hospitals and clinics offering unaffordable services. The modification of the present cervical cancer prevention and treatment programme is recommended. This should include:

1. Public health education should lay emphasis on the natural history of precancerous lesion and the importance of breaking the process.

2. Assuring women who test positive that cervical cancer is preventable and treatable if identified early.

3. Changing of the present multi-visit strategy to a single visit strategy of “see and treat” to ensure that all the women who test positive receive the lifesaving treatment.

4. Motivation of women to take up cervical cancer prevention and treatment services by making all cervical cancer services affordable if not completely free.

5. Decentralization of cervical cancer services to the rural areas using midwives and medical officers.

6. Integration of cervical cancer screening into existing reproductive health services like HIV and family planning programme.

## Competing interests

The authors declare that they have no competing interests.

## Authors’ contributions

OCE conceived, designed the study, supervised data collection, analyzed the data, drafted the paper and approved the final version. TAG, IEI and OK contributed to study design, collected the data and the draft of the paper. IAOU co-supervised the study, contributed to study design, reviewed result of data analysis and draft for important intellectual content. KOP and POO contributed to the conception, modified the original concept and design, supervised the study, reviewed and contributed to the manuscript drafts. All authors read and approved the final manuscript.

## Pre-publication history

The pre-publication history for this paper can be accessed here:

http://www.biomedcentral.com/1472-6963/14/143/prepub
